# Lumbar Spondylodiscitis Caused by Clostridium perfringens: A Case Report Highlighting Diagnostic Challenges and Conservative Management

**DOI:** 10.7759/cureus.81865

**Published:** 2025-04-08

**Authors:** Bruno Cancela, Dara Mbanze, João Miranda, Albina Moreira, Pedro Oliveira

**Affiliations:** 1 Physical Medicine and Rehabilitation, Centro de Reabilitação do Norte, Vila Nova de Gaia, PRT; 2 Infectious Diseases, Unidade Local de Saúde Gaia Espinho, Vila Nova de Gaia, PRT; 3 Internal Medicine, Unidade Local de Saúde Gaia Espinho, Vila Nova de Gaia, PRT

**Keywords:** anti-bacterial agents, clostridium perfringens, discitis, low back pain, spine, spondylitis

## Abstract

This case report describes a 70-year-old male with a history of hepatic cirrhosis and diabetes who was admitted with severe low back pain, fever, and sepsis. Initially, the back pain appeared consistent with a muscle strain. However, the persistence of symptoms, ongoing fever, and the patient’s underlying conditions raised suspicion for a more serious pathology. Blood cultures identified *Clostridium perfringens*, and MRI confirmed L4-L5 spondylodiscitis accompanied by an epidural abscess. This rare musculoskeletal infection, known for its high mortality risk, was successfully treated with targeted antibiotic therapy alone, as there were no neurological deficits to warrant surgical intervention. After nine weeks of treatment, a follow-up MRI showed the resolution of the abscess, and the patient made a full clinical recovery. The likely mechanism in this case is bacterial translocation driven by portal hypertension and immune dysregulation associated with liver cirrhosis. This case underscores the importance of investigating serious underlying causes in patients presenting with back pain and red flag symptoms, and it demonstrates that conservative management can be effective in spondylodiscitis without neurological involvement, even when caused by rare and high-risk pathogens such as *C. perfringens*.

## Introduction

*Clostridium perfringens *is an anaerobic, gram-positive bacillus commonly found in soil, water, and the human GI tract. It is most often associated with soft tissue infections (e.g., cellulitis and myonecrosis), acute GI infections, and septicemia [1]. The bacterium can cause disease through two primary mechanisms: traumatic and spontaneous [2]. Traumatic infections occur following injuries or wounds, such as crush injuries, compound fractures, penetrating trauma, or surgical procedures, where bacterial spores are directly introduced into the tissues [3]. In contrast, spontaneous infections develop in patients with underlying conditions such as malignancies or GI or liver diseases, without any apparent trauma, and are thought to result from bacterial translocation from the GI tract [4].

Here, we report a rare case of spondylodiscitis caused by *C. perfringens* in a 70-year-old patient with hepatic cirrhosis.

## Case presentation

A 70-year-old man presented to the ED with a one-week history of back pain and fever. The pain began after he lifted a heavy object and was localized in the lumbosacral region, approximately 3-4 cm left of the midline of the spine. It rapidly worsened during the day, with a pain rating of 9/10 on the Numerical Rating Scale. The back pain was accompanied by fever, with the highest measured temperature reaching 39 °C. The fever responded to paracetamol but recurred within less than eight hours. Relevant medical history included alcoholic hepatic cirrhosis (with portal hypertension), type 2 diabetes mellitus, arterial hypertension, and dyslipidemia.

On physical examination, the patient’s blood pressure was 84/47 mmHg, heart rate was 100 beats per minute, and temperature was 37.5 °C. He was jaundiced. The remainder of the physical examination, including the neurological evaluation, was normal. Blood gas analysis showed normal lactate levels, and initial laboratory findings, as shown in Table 1, revealed acute kidney injury, direct and indirect hyperbilirubinemia, elevated liver transaminases, alkaline phosphatase, gamma-glutamyl transferase, and C-reactive protein. Blood cultures were drawn, and fluid therapy along with empirical antimicrobial therapy (ceftriaxone and vancomycin) was initiated in the ED.

**Table 1:** Laboratory values at admission

Parameter	Value	Reference range
Total leukocyte count (×10³/µL)	10.57	3.8-10.6
Neutrophils (×10³/µL)	7.46	1.3-8.8
Eosinophils (×10³/µL)	0	0.0-0.7
Basophils (×10³/µL)	0.05	0.0-0.2
Lymphocytes (×10³/µL)	2.01	1.0-4.8
Monocytes (×10³/µL)	0.93	0.1-0.8
Creatinine (mg/dL)	2.52	0.61-1.17
Total bilirubin (mg/dL)	3.3	0.1-1.1
Direct bilirubin (mg/dL)	2.1	0.1-0.3
Aspartate aminotransferase (U/L)	91	4-33
Alanine aminotransferase (U/L)	230	4-50
Alkaline phosphatase (U/L)	151	40-129
Gamma-glutamyl transferase (U/L)	213	5-61
C-reactive protein (mg/dL)	18.45	0-0.5

**Figure 1:**
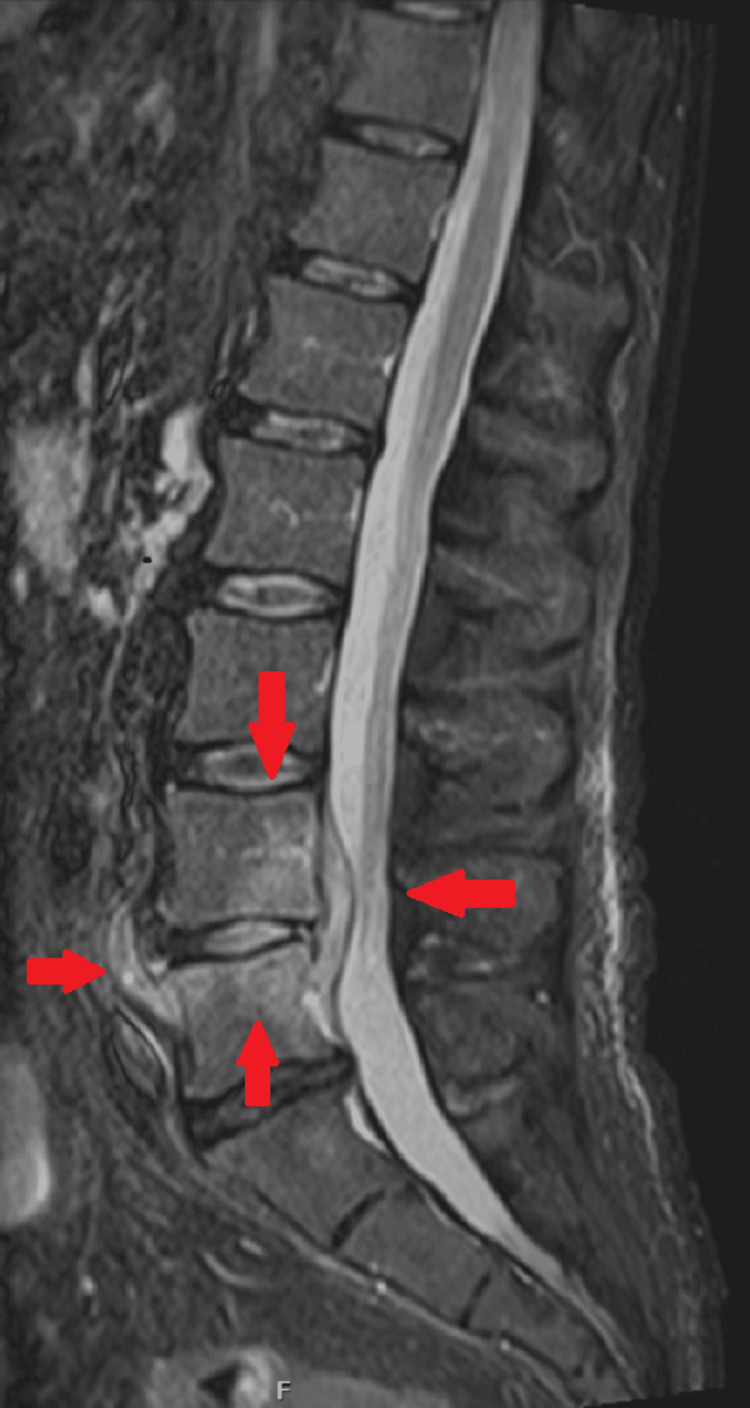
Initial lumbosacral MRI (STIR, sagittal) showing edema in the L4 and L5 vertebral bodies and an epidural abscess STIR, short tau inversion recovery

**Figure 2:**
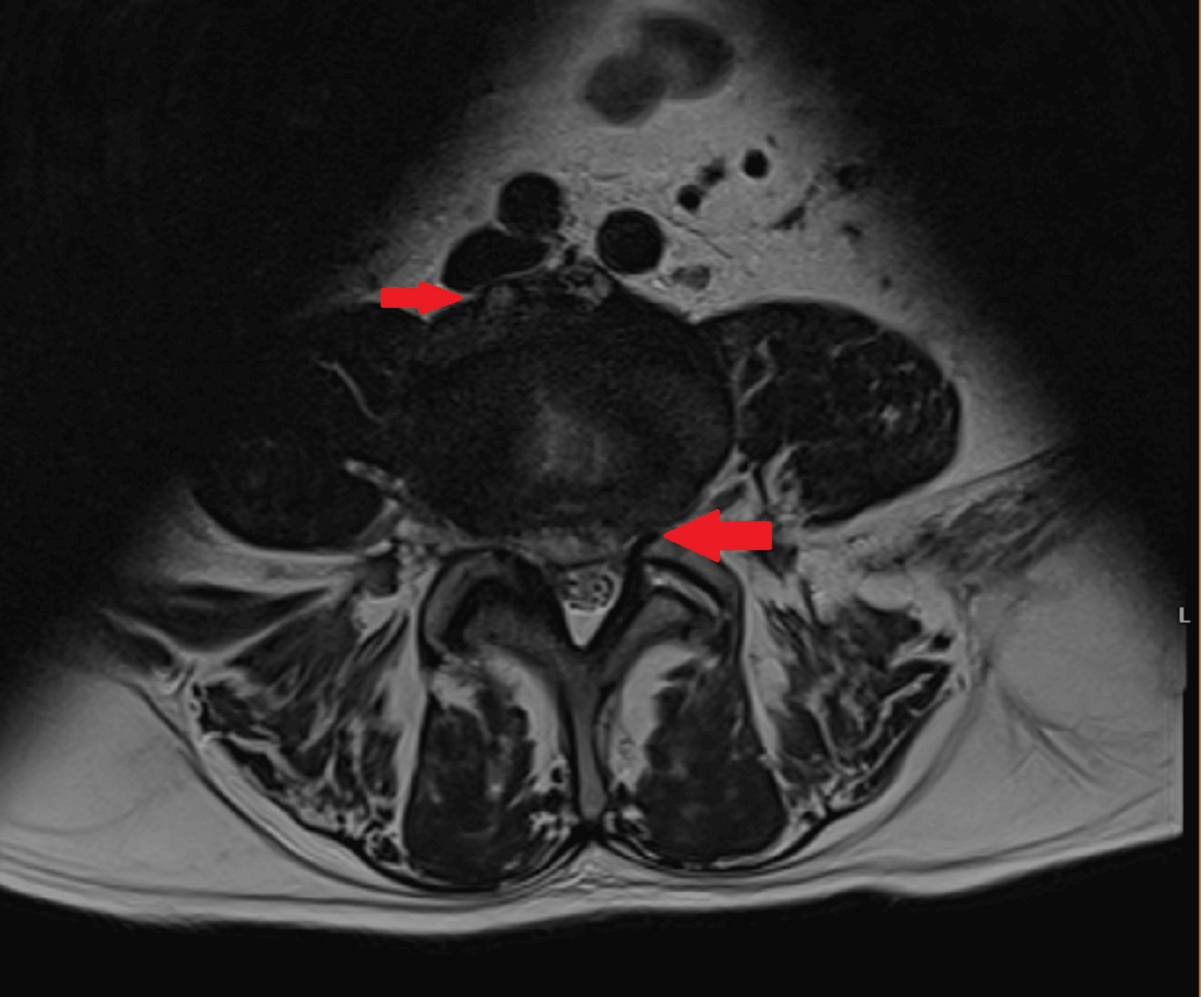
Initial lumbosacral MRI (T2, transversal) showing edema in the L4 and L5 vertebral bodies and an epidural abscess

The patient was admitted to the internal medicine ward for continued care. The back pain persisted despite treatment with paracetamol, tramadol, and morphine. Upon reevaluation of the pain area, muscle spasms were noted in a very painful region, likely affecting the left quadratus lumborum muscle. The neurological exam showed no abnormalities. Based on these findings, the previous analgesics were discontinued, and ketorolac and thiocolchicoside were introduced, leading to significant improvement (with pain rated at 1/10 on the NRS) after four days of treatment. The antimicrobial regimen (ceftriaxone and vancomycin) was maintained, but there was no improvement in fever or inflammatory markers. On the fourth day of hospitalization, blood cultures tested positive for *C. perfringens*, prompting a change in antimicrobial therapy to piperacillin-tazobactam and clindamycin. An echocardiogram was performed and was negative for vegetation or abscesses. Lumbosacral MRI (Figure 1, Figure 2) showed edema in the L4 and L5 vertebral bodies, along with associated anterior and posterior subligamentous tissue involvement. A non-capturing area on the posterior epidural component measured 60 mm (craniocaudally) by 6 mm (sagittally), indicating an epidural abscess, along with edema of the L4-L5 intervertebral disk. These findings confirmed the diagnosis of spondylodiscitis. After consultation with neurosurgery, surgical intervention was deemed unnecessary. Seven days after starting piperacillin-tazobactam and clindamycin, the patient became afebrile, the inflammatory markers progressively decreased, and follow-up blood cultures were negative.

**Figure 3:**
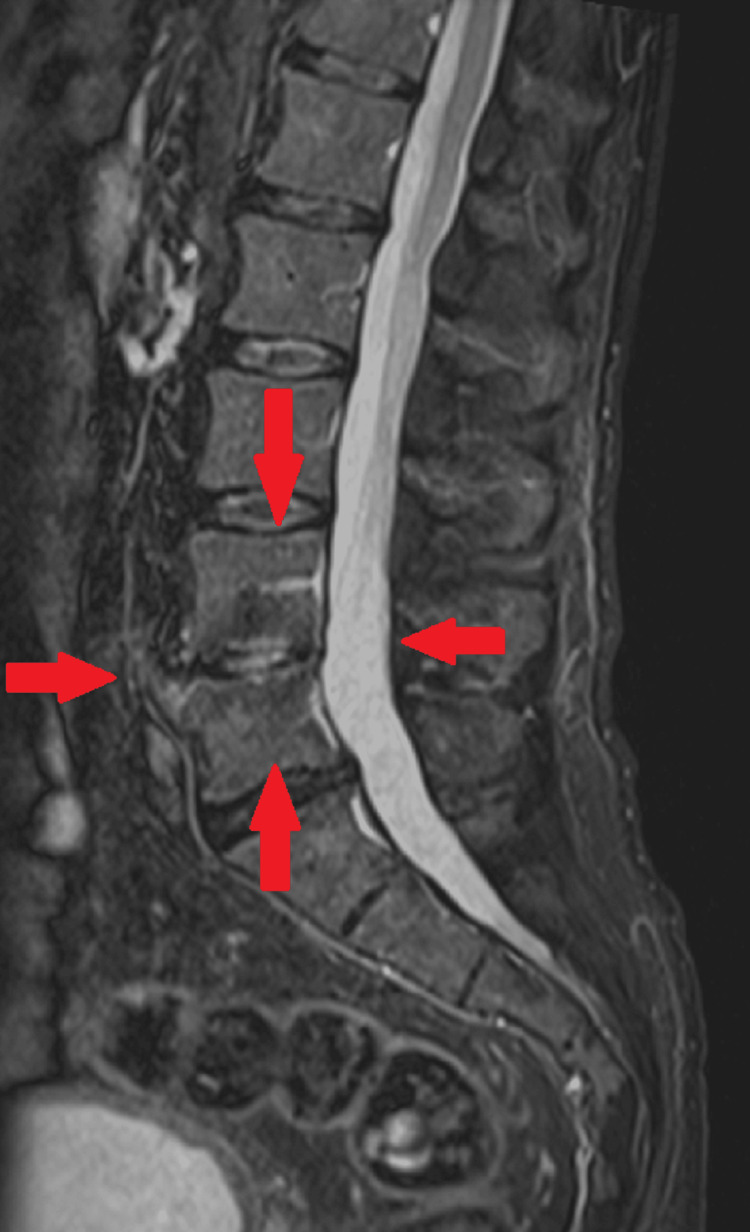
Follow-up (eight weeks) lumbosacral MRI (STIR, sagittal) showing resolution of the epidural collection STIR, short tau inversion recovery

**Figure 4:**
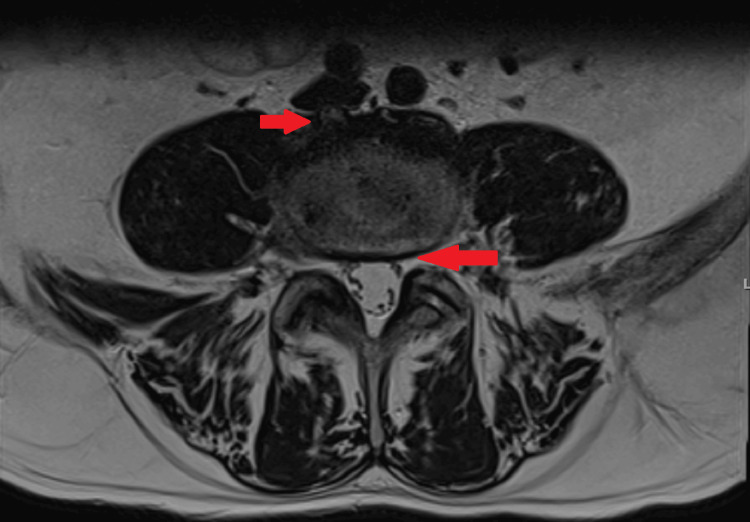
Follow-up (eight weeks) lumbosacral MRI (T2, transversal) showing resolution of the epidural collection

The patient continued the same antimicrobial regimen, completing a total of six weeks of treatment under domiciliary hospitalization with favorable clinical and analytical progress. Follow-up MRI at five weeks of treatment showed a reduction in the epidural collection, which now measured 50 mm (craniocaudally) by 3 mm (sagittally). Based on these findings, the antimicrobial regimen was switched to ceftriaxone and clindamycin, allowing the patient to complete nine weeks of treatment on an outpatient regimen. The symptoms (including fever and low back pain) did not recur, and a follow-up MRI at eight weeks (Figure 3, Figure 4) of treatment showed persistent edema in the L4 and L5 vertebral bodies but complete resolution of the epidural collection. At eight weeks of follow-up, a colonoscopy was recommended to the patient, which revealed no abnormalities.

## Discussion

We present a case of spondylodiscitis in a 70-year-old man who was admitted to the ER with sepsis (hepatic and renal dysfunction), fever, and lumbar pain. The organic dysfunction was rapidly resolved with initial management, including fluid resuscitation and antimicrobial therapy. The lumbar pain was initially interpreted as muscle strain because it started after an episode of increased physical activity, the physical examination was relatively normal, and the pain improved with non-steroidal anti-inflammatory drugs and a muscle relaxant. This case underscores the importance of considering the classic red flags for lumbar pain, which in this case included severe pain, fever, and the patient’s history of hepatic cirrhosis. This history may have caused immunosuppression, increasing the risk of an underlying malignancy such as hepatocellular carcinoma. These red flags prompted the consideration of spondylodiscitis as a differential diagnosis and led to further investigation using imaging studies like lumbar CT and MRI.

The clinical presentation and laboratory values raised suspicion of a spinal infection, which led to the initiation of empiric antimicrobial therapy. The vacuum phenomenon, characterized by gas accumulation within the intervertebral disc, is typically associated with degenerative disc disease [5], but it has also been observed in cases of spondylodiscitis caused by *C. perfringens* [6]. The diagnosis was confirmed by positive blood cultures and specific MRI findings. The decision not to perform a biopsy was based on the patient’s positive clinical progress and the absence of surgical indications. The differential diagnosis also included a possible skin and soft tissue infection by *C. perfringens*, but the patient did not present any inflammatory signs on the skin or palpable gas in the subcutaneous tissue, making this hypothesis unlikely. Despite its rarity, given the absence of other infection sites and the favorable imaging evolution, spondylodiscitis caused by *C. perfringens *was confirmed as the most likely diagnosis.

*C. perfringens* is a gram-positive, anaerobic bacterium commonly found in the GI microbiota [7]. It is most often associated with clostridial myonecrosis and enteritis [8]. Musculoskeletal infections are rare [9], and only six cases of spondylodiscitis caused by *C. perfringens *have been described as of the writing of this article [6]. Infections caused by *C. perfringens *and other *Clostridioides* species are typically associated with high mortality rates (up to 71% at three months), primarily due to myonecrosis with gas gangrene formation or bacteremia leading to hemolysis and organ failure [10]. Various mechanisms of infection have been proposed, including bacterial dissemination after spinal surgery and GI infection, as well as an association with chronic hepatitis in one case [6]. In this patient, a history of hepatic cirrhosis was noted, but there were no GI symptoms or history of spinal trauma or surgery, leaving the exact pathogenesis unclear. Bacteremia caused by *C. perfringens* is often linked to underlying malignancies, such as colorectal cancer, which was ruled out in this patient [11]. Liver cirrhosis could be a risk factor for *C. perfringens* bacteremia, as it is common in patients with this condition [12]. We hypothesize that portal hypertension, combined with gut microbiome dysfunction and immune response dysregulation due to liver cirrhosis, may have facilitated bacterial translocation, leading to bacteremia and subsequent spinal infection.

Treatment for spondylodiscitis typically involves antibiotics, either alone or in conjunction with surgery. Surgical indications include failure of conservative management, spinal mechanical instability, and compression of neurological structures [13,14]. In this case, the patient showed no neurological signs or significant compression, so we opted for antibiotic therapy alone, with monitoring for any new neurological symptoms and follow-up MRI. The selected antimicrobial regimen included piperacillin-tazobactam and clindamycin, which have been recommended for severe *C. perfringens *infections [15]. Clindamycin is particularly effective due to its ability to inhibit toxin synthesis [16]. Outpatient treatment was appropriate for this patient, facilitated by switching from piperacillin-tazobactam to ceftriaxone based on sensitivity data from blood cultures and published evidence of efficacy with other beta-lactam agents [17,18]. The patient showed favorable clinical progress, and a follow-up MRI after nine weeks of treatment revealed complete resolution of the epidural collection, indicating an excellent response to the chosen therapy. This case emphasizes the validity of using medical treatment alone for spondylodiscitis without neurological involvement, even when caused by rare and high-risk pathogens like *C. perfringens*, as long as the etiological agent is identified and its antibiotic sensitivity is known. In this case, the positive blood cultures negated the need for drainage of the epidural collection.

## Conclusions

This case report emphasizes key points to consider when dealing with lower back pain: the diagnostic approach in the ED, the importance of recognizing “red flag” symptoms and signs, the role of imaging and microbial studies in guiding treatment and antimicrobial therapy, and the possibility of managing the condition conservatively (including domiciliary hospitalization or outpatient regimens) when there are no neurological signs, even in infections caused by rare agents with high mortality rates.
